# Clinical Outcomes Post‐Transcatheter Aortic Valve Replacement in Patients With Hypertrophic Obstructive Cardiomyopathy

**DOI:** 10.1002/ccd.70201

**Published:** 2025-09-23

**Authors:** Muhammad Usman Almani, Vibhor Ahluwalia, Muhammad Yousuf, Jafar Alzubi, Mohammad Hamza, Raphael Bonita, Christian Witzke

**Affiliations:** ^1^ Division of Cardiology, Jefferson Einstein Hospital Thomas Jefferson University Philadelphia Pennsylvania USA; ^2^ Department of Internal Medicine Nazareth Hospital Philadelphia Pennsylvania USA; ^3^ Division of Medicine Mayo Clinic Rochester Minnesota USA; ^4^ Division of Cardiology Yale University Hospital New Haven Connecticut USA; ^5^ Division of Medicine Guthrie Medical Group Cortland New York USA

**Keywords:** hypertrophic obstructive cardiomyopathy, suicide left ventricle, transcatheter aortic valve replacement

## Abstract

**Background:**

Patients with hypertrophic obstructive cardiomyopathy (HOCM) were excluded from all major trials for transcatheter aortic valve replacement (TAVR). “Suicide left ventricle” occurring after TAVR is postulated to occur as a result of chronic pressure overload from fixed obstruction that is acutely relieved after the valve deployment resulting in worsening left ventricular outflow tract obstruction.

**Aims:**

The aim of this study was to determine the clinical and readmission outcomes in HOCM patients undergoing TAVR.

**Methods:**

Data was extracted from the National Inpatient and National Readmission 2016−2020 Databases. We performed multivariable Logistic regression analysis to determine the odds of in‐hospital mortality and other relevant clinical outcomes in patients undergoing TAVR with and without comorbid HOCM. Multivariable Cox regression analysis was used to assess 30‐day hospital readmission in HOCM patients undergoing TAVR.

**Results:**

We identified 296,670 patients who underwent TAVR, of whom 534 (0.2%) had comorbid HOCM. The patients undergoing TAVR with comorbid HOCM had more than threefold higher risk of in‐hospital mortality (adjusted odds ratio: 3.47, 95% CI 1.37–8.81, *p* = 0.009) when compared to patients without HOCM. Additionally, patients undergoing TAVR with comorbid HOCM had a higher risk of atrioventricular blocks, cardiogenic shock, acute kidney injury, and the need for intubation. There was no difference in readmission outcomes post‐TAVR among patients with and without HOCM.

**Conclusion:**

Our analysis of a large, real‐world cohort of patients undergoing TAVR showed that patients with HOCM have a significantly higher risk of in‐hospital mortality and procedural complications when compared to patients without HOCM.

## 1 Introduction

Hypertrophic obstructive cardiomyopathy (HOCM), a genetic disorder characterized by unexplained left ventricular hypertrophy, affects approximately 1 in 500 individuals and frequently involves dynamic left ventricular outflow tract (LVOT) obstruction [1]. Severe aortic stenosis (AS), a progressive narrowing of the aortic valve, is prevalent in approximately 3.4% of individuals over the age of 75 [2]. The coexistence of HOCM and AS presents unique therapeutic and management challenges, particularly in the transcatheter aortic valve replacement (TAVR)‐eligible population, due to the overlapping yet distinct mechanisms of the LVOT obstruction.

TAVR has revolutionized the management of severe AS, offering a less invasive alternative to surgical aortic valve replacement (SAVR). Initially developed for patients at high surgical risk, the PARTNER 1 trial demonstrated TAVR superiority over medical therapy in inoperable patients [3]. Subsequent trials, including CoreValve and PARTNER 2, expanded TAVR indications to intermediate‐risk patients, establishing its non‐inferiority to SAVR [4, 5]. More recently, the PARTNER 3 and Evolut Low Risk trials have confirmed the benefits of TAVR in low‐risk patients, with outcomes comparable or superior to SAVR [6, 7].

Despite these expanding indications for TAVR, patients with HOCM were systematically excluded from major randomized controlled trials, such as the PARTNER and CoreValve trials, due to the unique challenges posed by the complex HOCM hemodynamics. As a result, there is a critical unmet need for robust data on the safety and efficacy of TAVR in this population. Existing literature is confined to small case series and single‐center studies, often limited by sample size, lack of comparator groups, and selection bias, precluding definitive conclusions [8]. This significant void in the literature underscores the need for larger, real‐world investigations that evaluate TAVR outcomes in HOCM patients.

The coexistence of HOCM and AS presents unique clinical challenges for patients eligible for TAVR. In HOCM patients, the relief of fixed obstruction from AS can unmask or exacerbate dynamic LVOT obstruction, potentially leading to acute hemodynamic instability, a phenomenon termed “suicide left ventricle” [9]. This potential complication, coupled with anatomical differences such as septal hypertrophy that may complicate valve deployment, underscores the urgent need for further study on TAVR outcomes in this high‐risk cohort. Understanding these outcomes is essential to enhance patient selection, optimize interventional procedural strategies, and improve the success and safety of the TAVR procedure in HOCM patients.

## 2 Methods

### 2.1 Design and Data Source

This was a retrospective analysis involving adult HOCM patients who underwent TAVR in the US from 2016 to 2020. We extracted the data from the National Inpatient Sample (NIS) and the National Readmission Database (NRD). We used the NIS for analysis of patient characteristics and in‐hospital outcomes, and the NRD for readmission analysis. The NIS and NRD are part of the Healthcare Cost and Utilization Project (HCUP) that is sponsored by the Agency for Healthcare Research and Quality (AHRQ). The NIS is the largest all‐payer database of hospital inpatient stays in the United States. Whereas the NRD is drawn from HCUP State Inpatient Databases (SID) that contain reliable, verified patient linkage numbers that can be used to track a person across hospitals within a state, while adhering to strict privacy guidelines. Each discharge information includes deidentified elements such as patient demographics, payment source, hospital characteristics, principal diagnosis, secondary diagnoses, and procedural diagnoses among others. The NIS and NRD contain a weighted sample of hospitalizations, and this can be used to derive national estimates. The study was exempt from institutional board review approval as the NIS and NRD databases contain deidentified patient information.

### 2.2 Study Population

Eligible patients for this study included US adults aged ≥ 18 years who underwent TAVR between 2016 and 2020 in the United States. We used International Classification of Diseases, 10th Revision, Procedure Coding System (ICD‐10‐PCS) codes to identify patients who underwent TAVR as shown in Supporting Information S1: Table 1. For readmission analysis using the NRD database, admissions were excluded as an index admission if the hospitalization was elective, had missing data for age, sex, or in‐hospital mortality, if the patient died during the hospital stay, or was transferred to another acute care hospital. In the NRD, patient identifiers cannot be linked across the years; hence, patients who had an index hospitalization on December 1 or later in any given year were excluded from the 30‐day readmission analysis. Time to readmission was calculated by subtracting the length of stay of the index admission from the time between the index admission and the readmission. Planned/elective readmissions were excluded. Readmissions for nonspecific traumatic diagnoses were excluded using the NECODE. The NECODE provides a method of classifying injuries. The NECODE used for nonspecific traumatic readmission exclusion were ICD‐10 codes which are “S, T, V, and Y.”

### 2.3 Variables

Patient demographics including age, sex, primary insurance, race, and median neighborhood household income (income quartiles were identified referred to patients as 1‐low income, 2‐middle income, 3‐upper middle income, and 4‐high income) using the NIS and NRD variables. In 2020, quartile 1 reflected household income: ≤ $49,999; quartile 2: $50,000–$64,999; quartile 3: $65,000–$85,999; quartile 4: ≥ $86,000. We included hospital‐specific variables including hospital bed size, hospital teaching status, and location. Comorbidities were identified using diagnosis codes from the International Classification of Diseases, 10th Revision, Clinical Modification (ICD‐10‐CM) respective to the years (Supporting Information S1: Table 1). The comorbid conditions were present before the hospitalization and were not the primary cause of admission. We used the Charlson comorbidity index to assess the severity of comorbid conditions.

### 2.4 Outcome Measures

For the primary analysis, we assessed the rate of in‐hospital mortality for patients who underwent TAVR. Secondary analyses evaluated the risk of atrioventricular blocks, bundle branch blocks, cardiogenic shock, acute kidney injury, need for intubation, need for vasopressors, need for temporary mechanical circulatory support devices (including intra‐aortic balloon pump, Impella assist system, and veno‐arterial extracorporeal membrane oxygenation), bleeding complications, 30‐day all‐cause, and 30‐day heart failure (HF)‐specific readmissions.

### 2.5 Statistical Analysis

As per specific HCUP recommendations, we performed our analysis utilizing the HCUP STATA survey data analysis packages. The Student *t*‐test and the chi‐squared test were used to compare continuous and categorical variables, respectively. In accordance with HCUP, any sample size less than 11 has been reported as ≤ 10 to protect patient privacy.

We performed multivariable Logistic regression analysis to determine the odds ratio (OR) of in‐hospital mortality, atrioventricular blocks, bundle branch blocks, cardiogenic shock, acute kidney injury, need for intubation, need for vasopressors, need for temporary mechanical circulatory support devices, and intracranial or gastrointestinal (GI) bleeding in patients undergoing TAVR with and without comorbid HOCM. Additionally, we performed multivariable Cox regression analysis with all‐cause readmission and HF‐specific readmission within 30 days as the “failure event” and time from index hospitalization discharge to failure event as the “time to failure event” to determine the hazard ratio (HR) of readmission. HF‐specific readmission was defined as the HF being the primary diagnosis for readmission. Time to failure event was calculated by subtracting length of stay of index admission from time between index admission and the failure event.

All the analyses were adjusted for age, gender, insurance status, Charlson comorbidity index, and hospital characteristics. A two‐sided *p* < 0.05 was considered to represent statistical significance. All analyses were performed using STATA version 16.

## 3 Results

### 3.1 Baseline Characteristics

We identified 296,670 patients that underwent TAVR between 2016 and 2020, of which 534 (0.2%) patients had comorbid HOCM and 682 (0.2%) patients had hypertrophic cardiomyopathy (HCM) without obstructive phenotype. Baseline patient and hospital characteristics of patients undergoing TAVR with and without HOCM are presented in Table 1. Mean age of patients who had TAVR with comorbid HOCM was 79.3 years, mean CCI was 2.8, and 67.9% of the patients were female. In contrast, the mean age of patients who had TAVR without comorbid HOCM was 78.9 years, mean CCI was 3.0 and 44.5% of the patients were female. Patients with HOCM were more likely to have rheumatic valvular heart disease (20.7% vs. 10.8%), whereas patients without HOCM were more likely to have prior percutaneous coronary intervention (19.2% vs. 9.4%) and prior coronary artery bypass grafting (15.6% vs. 3.8%).

**Table 1:** Baseline characteristics of TAVR hospitalizations.

Baseline characteristics	Without HOCM (*n* = 296,136)	With HOCM (*n* = 534)	*p* value
Age (years), mean	78.9	79.3	0.624
Charlson comorbidity index, mean	3.0	2.8	0.340
LOS (days), mean	3.9	4.5	0.206
Weekend admission, *n* (%)	10,069 (3.4)	15 (2.8)	0.744
Female, *n* (%)	131,781 (44.5)	363 (67.9)	< 0.001
Race, *n* (%)			0.180
Caucasians	258,231 (87.2)	447 (83.8)	
African Americans	12,142 (4.1)	41 (7.6)	
Hispanics	14,215 (4.8)	20 (3.8)	
Median household income, *n* (%)			0.339
Low‐income quartile	62,781 (21.2)	91 (17.1)	
Middle‐income quartile	76,995 (26.0)	117 (21.9)	
Upper‐middle income quartile	78,772 (26.6)	178 (33.3)	
High income quartile	77,588 (26.2)	148 (27.7)	
Insurance, *n* (%)			0.972
Medicare	261,488 (88.3)	484 (90.6)	
Medicaid	4146 (1.4)	≤ 10	
Private insurance	23,395 (7.9)	42 (7.9)	
Hospital location and teaching status, *n* (%)			0.648
Rural	3554 (1.2)	≤ 10	
Urban nonteaching	27,540 (9.3)	35 (6.6)	
Urban teaching	265,042 (89.5)	494 (92.5)	
Hospital region, *n* (%)			0.004
Northeast	67,519 (22.8)	136 (25.5)	
Midwest	67,815 (22.9)	186 (34.9)	
South	100,686 (34.0)	111 (20.7)	
West	60,116 (20.3)	101 (18.9)	
Hospital bed size, *n* (%)			0.143
Small	21,322 (7.2)	30 (5.7)	
Medium	62,188 (21.0)	75 (14.1)	
Large	212,626 (71.8)	428 (80.2)	
Hypertension, *n* (%)	265,634 (89.7)	489 (91.5)	0.545
Dyslipidemia, *n* (%)	216,179 (73.0)	408 (76.4)	0.423
Diabetes mellitus, *n* (%)	111,643 (37.7)	171 (32.1)	0.224
Atrial fibrillation/flutter, *n* (%)	90,618 (30.6)	141 (26.4)	0.347
Obesity, *n* (%)	59,523 (20.1)	136 (25.5)	0.178
Peripheral arterial disease, *n* (%)	28,725 (9.7)	56 (10.4)	0.812
Ischemic stroke, *n* (%)	5330 (1.8)	≤ 10	0.495
COPD, *n* (%)	66,927 (22.6)	126 (23.6)	0.817
Major depressive disorder, *n* (%)	23,691 (8.0)	30 (5.7)	0.378
Protein energy malnutrition, *n* (%)	5330 (1.8)	≤ 10	0.977
Anemia, *n* (%)	94,764 (32.0)	186 (34.9)	0.526
Pulmonary hypertension, *n* (%)	32,871 (11.1)	56 (10.4)	0.808
Presence of pacemaker, *n* (%)	27,245 (9.2)	66 (12.3)	0.306
Presence of ICD, *n* (%)	7403 (2.5)	25 (4.7)	0.143
Prior MI, *n* (%)	36,425 (12.3)	45 (8.5)	0.233
Prior PCI, *n* (%)	56,858 (19.2)	50 (9.4)	0.011
Prior CABG, *n* (%)	46,197 (15.6)	20 (3.8)	< 0.001
Rheumatic valvular heart disease, *n* (%)	31,983 (10.8)	111 (20.7)	0.001
Presence of prosthetic valve, *n* (%)	8588 (2.9)	25 (4.7)	0.279

Abbreviations: CABG, coronary artery bypass graft; COPD, chronic obstructive pulmonary disease; HOCM, hypertrophic obstructive cardiomyopathy; ICD, implantable cardioverter defibrillator; LOS, length of stay; MI, myocardial infarction; PCI, percutaneous coronary intervention; TAVR, transcatheter aortic valve replacement.

### 3.2 In‐Hospital Clinical Outcomes

**Figure 1:**
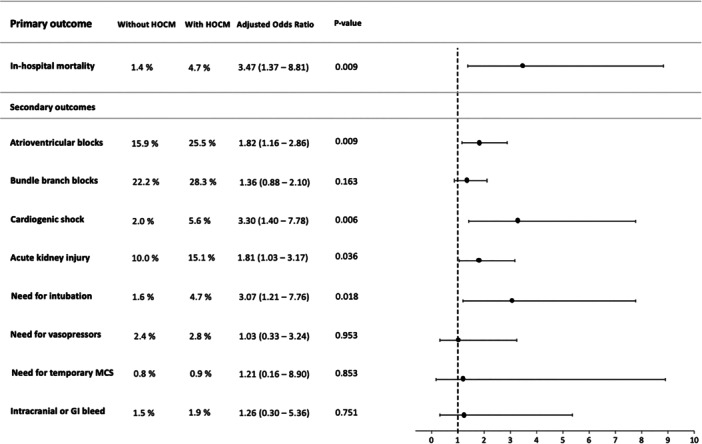
Clinical outcomes of patients with HOCM undergoing TAVR (reference group: Patients without HOCM). HOCM, hypertrophic obstructive cardiomyopathy, temporary; MCS, temporary mechanical circulatory support (which included intra‐aortic balloon pump, Impella assist system, and veno‐arterial extracorporeal membrane oxygenation).

The patients undergoing TAVR with comorbid HOCM had significantly higher risk of in‐hospital mortality (4.7% vs. 1.4%, adjusted OR: 3.47, 95% CI 1.32–8.81, *p* = 0.009), atrioventricular blocks (25.5% vs. 15.9%, adjusted OR: 1.82, 95% CI 1.16–2.86, *p* = 0.009), cardiogenic shock (5.6% vs. 2.0%, adjusted OR: 3.30, 95% CI 1.40−7.78, *p* = 0.006), acute kidney injury (15.1% vs. 10.0%, adjusted OR: 1.81, 95% CI 1.03–3.17, *p* = 0.036), need for intubation (4.7% vs. 1.6%, adjusted OR: 3.07, 95% CI 1.21–7.76, *p* = 0.018) compared to patients without HOCM. There was no difference in risk of bundle branch blocks (28.3% vs. 22.2%, adjusted OR: 1.36, 95% CI 0.88–2.10, *p* = 0.163), need for vasopressors (2.8% vs. 2.4%, adjusted OR: 1.03, 95% CI 0.33–3.24, *p* = 0.953), need for temporary MCS (0.9% vs. 0.8%, adjusted OR: 1.21, 95% CI 0.16–8.90, *p* = 0.853), and intracranial or GI bleeding (1.9% vs. 1.5%, adjusted OR: 1.26, 95% CI 0.30–5.36, *p* = 0.751) between the two groups (Figure 1).

Upon direct comparison, patients with HCM with LVOT obstruction who underwent TAVR showed higher, though not statistically significant, risk of in‐hospital mortality, atrioventricular blocks, bundle branch blocks, acute kidney injury, need for intubation, and intracranial or GI bleeding when compared to patients with HCM without LVOT obstruction as depicted in Supporting Information S1: Table 2.

### 3.3 30‐Day All‐Cause Readmission

**Figure 2:**
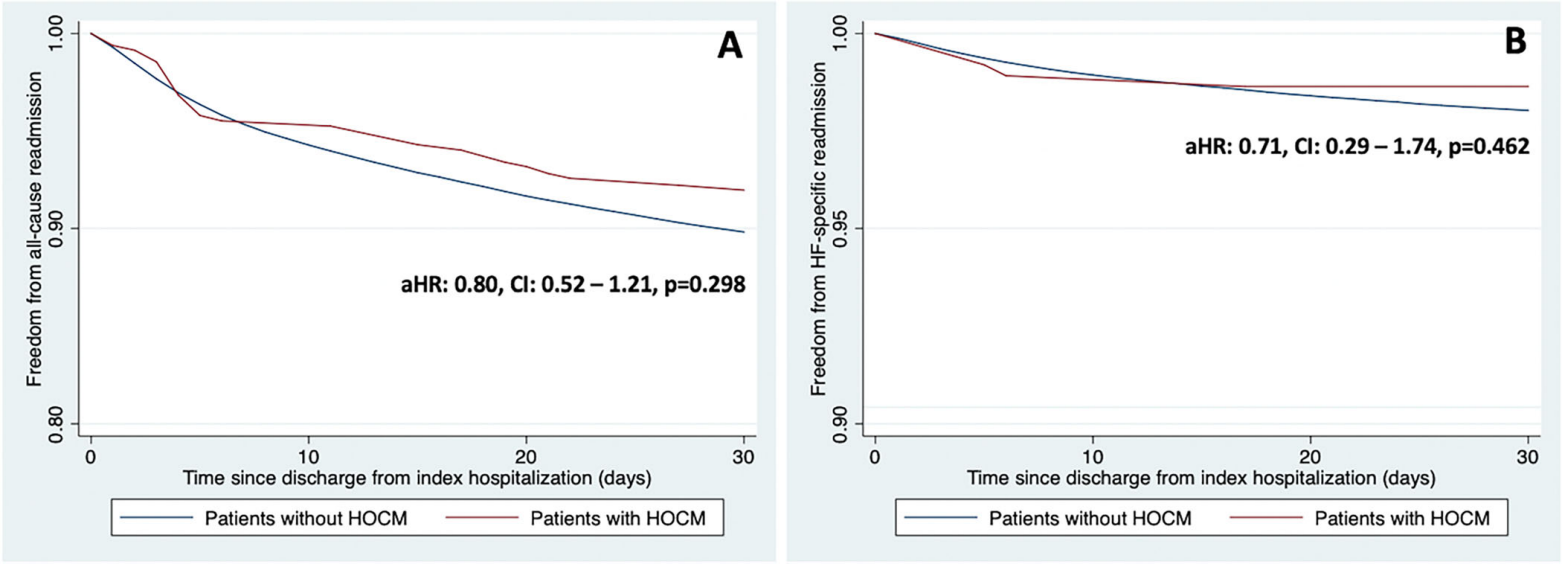
Readmission outcomes of patients with HOCM undergoing TAVR (reference group: Patients without HOCM). (A) Comparison of all‐cause readmission post‐TAVR. (B) Comparison of HF‐specific readmission post‐TAVR. aHR, adjusted hazard ratio; CI, confidence interval; HF, heart failure; HOCM, hypertrophic obstructive cardiomyopathy. [Color figure can be viewed at wileyonlinelibrary.com]

Among 287,573 patients analyzed for 30‐day all‐cause readmission, 10.2% of patients without HOCM and 8.0% of patients with HOCM were readmitted for any cause within 30 days of hospital discharge from index hospitalization (adjusted HR: 0.80, 95% CI 0.52–1.21, *p* = 0.298) as shown in Figure 2, panel A.

### 3.4 30‐Day HF‐Specific Readmission

Among 287,573 patients analyzed for 30‐day HF‐specific readmission, 2.0% of patients without HOCM and 1.4% of patients with HOCM were readmitted primarily for HF within 30 days of hospital discharge from index hospitalization (adjusted HR: 0.71, 95% CI 0.29–1.74, *p* = 0.462) as mentioned in Figure 2, panel B.

## 4 Discussion

The present study analyzed data from 296,670 patients undergoing TAVR between 2016 and 2020, of whom 0.2% (*n* = 534) had comorbid HOCM. Results of our study suggest that patients with HOCM undergoing TAVR experienced significantly higher risk of in‐hospital mortality (4.7% vs. 1.4%, adjusted OR: 3.47, 95% CI 1.32–8.81, *p* = 0.009), atrioventricular blocks (25.5% vs. 15.9%, adjusted OR: 1.82, 95% CI 1.16–2.86, *p* = 0.009), cardiogenic shock (5.6% vs. 2.0%, adjusted OR: 3.30, 95% CI 1.40–7.78, *p* = 0.006), acute kidney injury (15.1% vs. 10.0%, adjusted OR: 1.81, 95% CI 1.03–3.17, *p* = 0.036), need for intubation (4.7% vs. 1.6%, adjusted OR: 3.07, 95% CI 1.21–7.76, *p* = 0.018) compared to patients without HOCM. However, 30‐day all‐cause and HF‐specific readmission rates did not differ between the two groups (adjusted HR: 0.80, 95% CI: 0.52–1.21, *p* = 0.298 for all‐cause; adjusted HR: 0.71, 95% CI: 0.29–1.74, *p* = 0.462 for HF‐specific).

The higher in‐hospital mortality and complication rates of HOCM patients undergoing TAVR are likely attributable to the unique pathophysiological characteristics of HOCM [1] and their interaction with the hemodynamic changes induced by the TAVR procedure. In HOCM, dynamic LVOT obstruction, caused by septal hypertrophy and systolic anterior motion of the mitral valve, is the defining feature. The sudden relief of AS via TAVR reduces afterload, which can paradoxically exacerbate the LVOT gradient, leading to acute hemodynamic instability, a condition often termed “suicide left ventricle” [9]. Beyond LVOT dynamics, the marked septal hypertrophy (typically ≥ 15 mm, with severe cases exceeding 30 mm) and potential myocardial disarray characteristic of HOCM can create distinct anatomical challenges for TAVR. These may include difficulties in achieving optimal valve positioning and stable anchoring, an increased risk of paravalvular leakage if a uniform seal is impeded by the asymmetric septum, and a greater need for precise deployment to avoid iatrogenic injury to the conduction system, given its proximity to the hypertrophied septum. Moreover, the hypertrophied and often fibrotic myocardium in HOCM patients may be inherently more vulnerable to direct procedural trauma and subsequent conduction disturbances [10]. These factors likely contribute to the higher incidence of cardiogenic shock (5.6% vs. 2.0%, adjusted OR: 3.30, 95% CI 1.40–7.78, *p* = 0.006) and atrioventricular blocks (25.5% vs. 15.9%, adjusted OR: 1.82, 95% CI 1.16–2.86, *p* = 0.009) as observed in our study. The higher rates of acute kidney injury (adjusted OR: 1.81, 95% CI 1.03–3.17, *p* = 0.036) could be a consequence of hemodynamic perturbations, contrast‐induced nephropathy, or other procedural factors, as suggested by previous research [11]. Prior studies utilizing the NIS database found that HCM was associated with a significantly higher adjusted OR for in‐hospital mortality and other procedural complications [11, 12]. However, the aforementioned studies did not distinguish HCM patients with LVOT obstruction from HCM patients without LVOT obstruction. We found a higher, though not statistically significant, risk of in‐hospital mortality, atrioventricular blocks, bundle branch blocks, acute kidney injury, need for intubation, and intracranial or GI bleeding in HCM patients with LVOT obstruction who underwent TAVR when compared to their counterparts without LVOT obstruction. The higher trend of adverse outcomes in HCM patients with LVOT obstruction suggests that the two HCM phenotypes may behave differently in terms of TAVR procedural outcomes.

In contrast to the elevated in‐hospital complications observed in HOCM patients undergoing TAVR, our study found no significant difference in 30‐day all‐cause (adjusted HR: 0.80, 95% CI 0.52–1.21, *p* = 0.298) and HF‐specific readmission rates (adjusted HR: 0.71, 95% CI 0.29–1.74, *p* = 0.462) compared to patients without HOCM. This finding is surprising given the higher rates of in‐hospital mortality and complications in the HOCM cohort. Several factors may explain this observation. Effective management of peri‐procedural complications during the index hospitalization likely stabilizes patients sufficiently to prevent early readmissions. For instance, addressing hemodynamic instability or conduction disturbances before discharge may mitigate short‐term risks [13, 14]. Additionally, standardized post‐discharge care protocols are likely applied uniformly to TAVR patients, potentially equalizing readmission risks across various at‐risk groups. It is also possible that a selection effect occurs, where only HOCM patients who tolerate the procedure well are discharged, thus reducing their readmission risk to levels comparable with non‐HOCM patients. These findings suggest that with appropriate peri‐procedural and post‐discharge management, HOCM patients who survive to discharge can achieve readmission rates comparable to those without HOCM.

To our knowledge, this is the largest study to date evaluating outcomes of TAVR in patients with HOCM. Our study highlights the need for meticulous perioperative management to improve outcomes in this high‐risk population. A thorough preprocedural assessment is essential, particularly to evaluate for LVOT obstruction and risk factors for obstructive physiology. For carefully selected patients with significant LVOT obstruction, pre‐TAVR interventions such as alcohol septal ablation (ASA) may reduce this risk by decreasing septal thickness, facilitating safer valve implantation [15]. Patient selection for ASA in this context typically considers the degree of septal hypertrophy, the severity of LVOT obstruction, and overall patient candidacy for septal reduction therapy. During the TAVR procedure, preparedness for conduction disturbances is critical. The increased incidence of atrioventricular blocks in HOCM patients necessitates having temporary pacing available and readiness for permanent pacemaker implantation if required. Similarly, the elevated risk of cardiogenic shock underscores the importance of having temporary mechanical circulatory support readily available to manage hemodynamic instability if it were to occur. After TAVR, close hemodynamic monitoring is vital, especially immediately after valve deployment, to promptly detect and address complications. Intraprocedural transesophageal echocardiography should be employed to assess the LVOT gradient and ensure no significant obstruction persists [16].

This study has several limitations that warrant consideration. The NIS and NRD database studies are subject to all the biases associated with retrospective analyses including selection, confounding, and measurement biases. Additionally, these administrative databases lack granular clinical data, such as the severity of HOCM, the degree of LVOT obstruction, and specific procedural details of TAVR, all of which could influence outcomes. The sample size of HOCM patients (*n* = 534) may be insufficient to detect differences in readmission rates, particularly after exclusions for the readmission analysis.

Despite these limitations, our study offers critical insights into TAVR outcomes in HOCM patients, a population underrepresented in clinical research. Given their exclusion from major TAVR trials [3, 4, 5, 6, 7] and the acknowledgment in current guidelines of limited data on TAVR in this group [17], there is a pressing need for dedicated studies. Future research should prioritize prospective studies or registries that incorporate detailed clinical and echocardiographic data to elucidate the risks and benefits of TAVR in HOCM patients. While there are published studies comparing outcomes of TAVR versus SAVR in HOCM patients, they are subject to significant selection bias and unmeasured confounding, preventing definitive conclusions about the optimal treatment strategy. Mhanna et al. published a NIS database analysis reporting a lower in‐hospital mortality, reduced rates of bleeding requiring transfusion, AKI, vascular complications, and shorter hospital stay with TAVR compared to SAVR in HCM patients with severe AS [18]. Furthermore, a systematic review of 11 studies similarly noted short‐term benefits for TAVR in this patient population but stressed the need for randomized trials due to inherent biases [19]. Given the heterogeneity of HOCM, stratifying patients by the presence and severity of LVOT obstruction and other structural and anatomical features may elucidate stratification of risk and inform personalized management approaches. Standardized definitions and reporting of HOCM characteristics within TAVR registries would also be beneficial for future clinical outcomes research.

## Conflicts of Interest

The authors declare no conflicts of interest.

## Supporting information

Online Supplement.

## Data Availability

The national inpatient sample (NIS) and national readmission database (NRD) are readily and publicly available. The patient information is deidentified, thus exempting the studies from the IRB process. URL: https://hcup-us.ahrq.gov/databases.jsp.
