# Supplementary material for: Clinical Outcomes Post‐Transcatheter Aortic Valve Replacement in Patients With Hypertrophic Obstructive Cardiomyopathy
Source: Catheter Cardiovasc Interv. 2025 Sep 23;106(7):3419–26. doi: 10.1002/ccd.70201 (PMC12679522; doi:10.1002/ccd.70201)
Supplement: Supplementary file 1 — Online Supplement. [file CCD-106-3419-s001.docx]

**Supplemental Table 1. Diagnosis Codes Utilized in Study**

| **Condition** | **ICD-10 CM/PCS Codes** |
| --- | --- |
| Transcatheter aortic valve replacement | 02RF37Z, 02RF38Z, 02RF3JZ, 02RF3KZ, 02RF37H, 02RF38H, 02RF3JH, 02RF3KH |
| Hypertrophic Cardiomyopathy with LVOT obstruction | I421 |
| Hypertrophic Cardiomyopathy without LVOT obstruction | I422 |
| Atrioventricular blocks | I440, I441, I442, I4430, I4439 |
| Bundle branch blocks | I444, I445, I4460, I4469, I447, I450, I4510, I4519, I452, I453 |
| Cardiogenic shock | R570 |
| Acute kidney injury | N17 |
| Need for intubation | 0BH17EZ |
| Need for vasopressors | 3E030XZ, 3E033XZ, 3E040XZ, 3E043XZ |
| Need for temporary MCS | 5A0221D, 5A0211D, 5A1522G, 5A02210, 5A02110 |
| Intracranial or GI bleed | I60, I61, I62, S064, K2081, K2091, K2101, K2211, K250, K252, K254, K256, K260, K262, K264, K266, K270, K272, K274, K276, K280, K282, K284, K286, K2901, K2921, K2931, K2941, K2951, K2961, K2971, K2981, K2991, K31811, K3182, K920, K921, K922, I8501, I8511, K5521, K625, K5701, K5711, K5721, K5731, K5741, K5751, K5781, K5791, K5713, K5733, K5753, K5793, K6381 |
| Hypertension | I10, I110, I119, I120, I129, I130, I1310, I1311, I132, I150, I151, I152, I158, I159 |
| Diabetes mellitus | E08–E13 |
| Obesity | E66 |
| Dyslipidemia | E78 |
| Atrial fibrillation/flutter | I48 |
| Peripheral arterial disease | I739 |
| COPD | J41, J42, J43, J44 |
| Pulmonary hypertension | I270, I272 |
| Ischemic stroke | I63 |
| Major depressive disorder | F32, F33 |
| Protein energy malnutrition | E43, E440, E441 |
| Anemia | D50–D64 |
| Presence of pacemaker | Z950 |
| Presence of ICD | Z95810 |
| Prior coronary artery bypass grafting | Z951 |
| Prior percutaneous coronary intervention | Z955 |
| Prior myocardial infarction | I252 |
| Rheumatic valvular disease | I05–I08 |
| Presence of prosthetic valve | Z952, Z953 |

LVOT: left ventricular outflow tract, MCS: mechanical circulatory support, GI: gastrointestinal, COPD: chronic obstructive pulmonary disease, ICD: implantable cardioverter defibrillator.

**Supplemental Table 2. Clinical outcomes of HCM patients with LVOTO undergoing TAVR compared to HCM patients without LVOTO (reference group: HCM patients without LVOTO)**

| **Outcome** | **HCM patients without LVOTO, %** | **HCM patients with LVOTO, %** | **aOR (95% CI)** | **p-value** |
| --- | --- | --- | --- | --- |
| Primary outcome | | | | |
| In-hospital mortality | 2.9 | 4.7 | 1.61 (0.38 – 6.78) | 0.512 |
| Secondary outcomes | | | | |
| Atrioventricular blocks | 20.9 | 25.5 | 1.18 (0.63 – 2.23) | 0.600 |
| Bundle branch blocks | 25.9 | 28.3 | 1.16 (0.65 – 2.06) | 0.605 |
| Cardiogenic shock | 6.5 | 5.6 | 0.91 (0.29 – 2.91) | 0.879 |
| Acute kidney injury | 13.0 | 15.1 | 1.34 (0.58 – 3.13) | 0.491 |
| Need for intubation | 2.2 | 4.7 | 2.16 (0.46 – 10.09) | 0.328 |
| Need for vasopressors | 3.6 | 2.8 | 0.90 (0.17 – 4.84) | 0.902 |
| Need for temporary MCS | 2.9 | 0.9 | 0.38 (0.04 – 3.42) | 0.386 |
| Intracranial or GI bleeding | 0.7 | 1.9 | 4.64 (0.34 – 63.18) | 0.249 |

HCM: hypertrophic cardiomyopathy, LVOTO: left ventricular outflow tract obstruction, aOR: adjusted odds ratio, MCS: mechanical circulatory support, GI: gastrointestinal.
